# (*E*,*E*)-1-Methyl-2,6-distyrylpyridinium iodide

**DOI:** 10.1107/S1600536810018519

**Published:** 2010-06-26

**Authors:** Narsimha Reddy Penthala, Joshua Eldridge, Thirupathi Reddy Yerram Reddy, Sean Parkin, Peter A. Crooks

**Affiliations:** a Department of Pharmaceutical Sciences, College of Pharmacy, University of Kentucky, Lexington, KY 40536, USA; b Department of Chemistry, University of Kentucky, Lexington, KY 40506, USA

## Abstract

In the title compound, C_22_H_20_N^+^·I^−^, the dihedral angles between the central pyridine ring and two outer benzene rings are 15.30 (10) and 11.82 (11)°. There are inter­molecular π–π stacking inter­actions between the nearest phenyl ring over an inversion-related pyridyl ring, the shortest centroid–centroid distance being 3.672 (3) Å. The crystal structure of the compound indicates the 2,6-distyryl substituents have an *E* configuration.

## Related literature

For the conventional synthesis, see: Stanek *et al.* (1952 ▶). For the activity of related compounds, see Zheng *et al.* (2005 ▶).
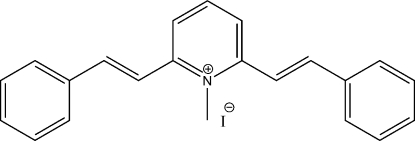


## Experimental

### 

#### Crystal data

C_22_H_20_N^+^·I^−^
*M* *_r_* = 425.29
Monoclinic, 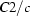

*a* = 18.7309 (4) Å
*b* = 9.5687 (2) Å
*c* = 19.9829 (4) Å
β = 90.279 (1)°
*V* = 3581.50 (13) Å^3^
*Z* = 8
Cu *K*α radiation
μ = 14.04 mm^−1^
*T* = 90 K
0.18 × 0.14 × 0.08 mm

#### Data collection

Bruker X8 Proteum diffractometer
Absorption correction: multi-scan (*SADABS*; Bruker, 2001 ▶) *T*_min_ = 0.211, *T*_max_ = 0.400
24972 measured reflections
3303 independent reflections
3286 reflections with *I* > 2σ(*I*)
*R*_int_ = 0.043

#### Refinement

*R*[*F* ^2^ > 2σ(*F* ^2^)] = 0.021
*wR*(*F* ^2^) = 0.055
*S* = 1.09
3303 reflections
219 parameters
H-atom parameters constrained
Δρ_max_ = 0.58 e Å^−3^
Δρ_min_ = −0.49 e Å^−3^

### 

Data collection: *APEX2* (Bruker, 2007 ▶); cell refinement: *SAINT* (Bruker, 2007 ▶); data reduction: *SAINT*; program(s) used to solve structure: *SHELXTL* (Sheldrick, 2008 ▶); program(s) used to refine structure: *SHELXTL*; molecular graphics: *SHELXTL*; software used to prepare material for publication: *SHELXTL* and local procedures.

## Supplementary Material

Crystal structure: contains datablocks global, I. DOI: 10.1107/S1600536810018519/nk2028sup1.cif

Structure factors: contains datablocks I. DOI: 10.1107/S1600536810018519/nk2028Isup2.hkl

Additional supplementary materials:  crystallographic information; 3D view; checkCIF report

## Figures and Tables

**Fig. 1.**
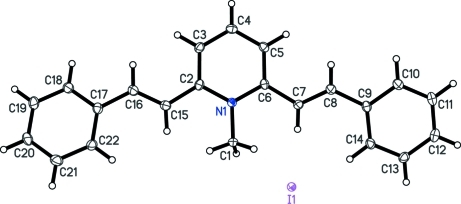
A view of the molecule with the atom numbering scheme. Displacement ellipsoids are drawn at the 50% probability level.

## supplementary crystallographic information

### Comment

In continuation of our work on the lobeline analogues, and structure-affinity relationships of novel ligands for the vesicular monoamine transporter (Zheng *et al.*, 2005), we have undertaken the design, synthesis and structural analysis of a series of phenyl substituted *N*-alkyl distyrylpyridine analogs. The primary reasons for the X-ray analysis of the title compound was to confirm the geometry of 2,6-distyryl groups and to obtain detailed information on the molecular structure. This information will be useful in structure-activity relationship (SAR) studies. The title compound was prepared by the reaction of *N*-methyl, 2,6-lutidine iodide with benzaldehyde in the presence of pyrrolidine in ethanol under microwave irradiation at 25-29 W power level and at 130 °C for 3 minutes (Biotage microwave initiator). The compound was recrystallized from ethanol. The molecular structure and the atom-numbering scheme are shown in Fig.1. The X-ray studies revealed that the 2,6-distyryl substituents in the title compound both have *E* geometry. The central pyridine ring makes a dihedral angle of 15.30 (10)° and 11.82 (11)° with the adjacent phenyl rings.

### Experimental

A mixture of *N*-methyl, 2,6-lutidine iodide (0.249 g, 1.0 mmol), benzaldehyde (0.300 g, 2.5 mmol) and pyrrolidine (6 µl) in ethanol (2 ml) was placed in a microwave-ready pressure vial equipped with a stirbar and irradiated in a Biotage microwave initiator for 3 minutes with the temperature set at 130 °C, at a power range of 25-29 W at 5 bar pressure. The cooled reaction mixture was taken out of the initiator, diluted with ethyl acetate, and filtered to afford a crude yellow solid. Crystallization from alcohol produced a yellow crystalline product of *N*-methyl-2,6-(*E*)distyrylpyridinium iodide that was suitable for X-ray analysis. ^1^H NMR (DMSO d_6_): δ 4.28 (*s*, 3H), 7.47-7.49 (*d*, *J*=6 Hz, 4H), 7.62-7.27 (*dd*, *J*=30.3 Hz, *J*=15.9 Hz, 6H), 7.848-7.872 (*d*, *J*=7.2 Hz, 4H), 8.26-8.29 (*m*, 2H), 8.39-8.44 (*t*, *J*=7.8 Hz, 1H); ^13^C NMR (DMSO d_6_): δ 41.97, 119.09, 124.02, 128.40, 128.91, 130.39, 134.87, 142.22, 143.11, 149.88, 153.10.

### Refinement

H atoms were found in difference Fourier maps and subsequently placed in idealized positions with constrained distances of 0.98 Å (RCH_3_), 0.95 Å (C_Ar_H), and with U_iso_(H) values set to either 1.2U_eq_ or 1.5U_eq_ (RCH_3_) of the attached atom.

### Crystal data

**Table:**

C_22_H_20_N^+^·I^−^	*F*(000) = 1696
*M**_r_* = 425.29	*D*_x_ = 1.577 Mg m^−^^3^
Monoclinic, *C*2/*c*	Cu *K*α radiation, λ = 1.54178 Å
Hall symbol: -C 2yc	Cell parameters from 9883 reflections
*a* = 18.7309 (4) Å	θ = 4.4–68.5°
*b* = 9.5687 (2) Å	µ = 14.04 mm^−^^1^
*c* = 19.9829 (4) Å	*T* = 90 K
β = 90.279 (1)°	Shard, yellow
*V* = 3581.50 (13) Å^3^	0.18 × 0.14 × 0.08 mm
*Z* = 8	

### Data collection

**Table:**

Bruker X8 Proteum diffractometer	3303 independent reflections
Radiation source: fine-focus rotating anode	3286 reflections with *I* > 2σ(*I*)
graded multilayer optics	*R*_int_ = 0.043
Detector resolution: 5.6 pixels mm^-1^	θ_max_ = 68.5°, θ_min_ = 4.4°
φ and ω scans	*h* = −22→22
Absorption correction: multi-scan (*SADABS* in *APEX2*; Bruker, 2001)	*k* = −11→11
*T*_min_ = 0.211, *T*_max_ = 0.400	*l* = −24→24
24972 measured reflections	

### Refinement

**Table:**

Refinement on *F*^2^	Secondary atom site location: difference Fourier map
Least-squares matrix: full	Hydrogen site location: inferred from neighbouring sites
*R*[*F*^2^ > 2σ(*F*^2^)] = 0.021	H-atom parameters constrained
*wR*(*F*^2^) = 0.055	*w* = 1/[σ^2^(*F*_o_^2^) + (0.0309*P*)^2^ + 5.1348*P*] where *P* = (*F*_o_^2^ + 2*F*_c_^2^)/3
*S* = 1.09	(Δ/σ)_max_ = 0.003
3303 reflections	Δρ_max_ = 0.58 e Å^−^^3^
219 parameters	Δρ_min_ = −0.49 e Å^−^^3^
0 restraints	Extinction correction: *SHELXTL* (Sheldrick, 2008), Fc^*^=kFc[1+0.001xFc^2^λ^3^/sin(2θ)]^-1/4^
Primary atom site location: structure-invariant direct methods	Extinction coefficient: 0.000067 (15)

### Special details

**Table:**

Geometry. All esds (except the esd in the dihedral angle between two l.s. planes) are estimated using the full covariance matrix. The cell esds are taken into account individually in the estimation of esds in distances, angles and torsion angles; correlations between esds in cell parameters are only used when they are defined by crystal symmetry. An approximate (isotropic) treatment of cell esds is used for estimating esds involving l.s. planes.
Refinement. Refinement of *F*^2^ against ALL reflections. The weighted *R*-factor wR and goodness of fit *S* are based on *F*^2^, conventional *R*-factors *R* are based on *F*, with *F* set to zero for negative *F*^2^. The threshold expression of *F*^2^ > 2σ(*F*^2^) is used only for calculating *R*-factors(gt) etc. and is not relevant to the choice of reflections for refinement. *R*-factors based on *F*^2^ are statistically about twice as large as those based on *F*, and *R*- factors based on ALL data will be even larger.

### Fractional atomic coordinates and isotropic or equivalent isotropic displacement parameters (Å^2^)

**Table:**

	*x*	*y*	*z*	*U*_iso_*/*U*_eq_	
I1	0.157566 (6)	0.613743 (12)	0.396017 (5)	0.01533 (8)	
N1	0.38671 (8)	0.46130 (16)	0.46816 (7)	0.0115 (3)	
C1	0.36127 (12)	0.60507 (19)	0.45527 (12)	0.0192 (5)	
H1A	0.4014	0.6634	0.4410	0.029*	
H1B	0.3407	0.6437	0.4963	0.029*	
H1C	0.3248	0.6034	0.4199	0.029*	
C2	0.44830 (10)	0.4136 (2)	0.43884 (9)	0.0133 (4)	
C3	0.47346 (10)	0.2818 (2)	0.45649 (9)	0.0153 (4)	
H3	0.5164	0.2477	0.4374	0.018*	
C4	0.43651 (10)	0.2005 (2)	0.50154 (9)	0.0146 (4)	
H4	0.4544	0.1112	0.5139	0.018*	
C5	0.37368 (10)	0.24873 (19)	0.52856 (9)	0.0136 (4)	
H5	0.3477	0.1915	0.5588	0.016*	
C6	0.34807 (11)	0.37993 (18)	0.51204 (10)	0.0120 (4)	
C7	0.28002 (10)	0.4316 (2)	0.53757 (9)	0.0133 (4)	
H7	0.2554	0.5026	0.5135	0.016*	
C8	0.25134 (11)	0.38157 (18)	0.59407 (10)	0.0146 (4)	
H8	0.2794	0.3177	0.6194	0.018*	
C9	0.18083 (10)	0.4165 (2)	0.62012 (9)	0.0139 (4)	
C10	0.15174 (11)	0.3314 (2)	0.67007 (10)	0.0177 (4)	
H10	0.1795	0.2576	0.6884	0.021*	
C11	0.08281 (11)	0.3536 (2)	0.69312 (10)	0.0191 (4)	
H11	0.0636	0.2945	0.7267	0.023*	
C12	0.04199 (10)	0.4616 (2)	0.66730 (10)	0.0180 (4)	
H12	−0.0055	0.4758	0.6823	0.022*	
C13	0.07150 (11)	0.5492 (2)	0.61904 (9)	0.0169 (4)	
H13	0.0442	0.6250	0.6021	0.020*	
C14	0.13969 (10)	0.5276 (2)	0.59548 (9)	0.0154 (4)	
H14	0.1588	0.5882	0.5625	0.018*	
C15	0.48534 (11)	0.5004 (2)	0.38947 (10)	0.0189 (4)	
H15	0.4637	0.5858	0.3760	0.023*	
C16	0.54716 (11)	0.4660 (2)	0.36280 (10)	0.0183 (4)	
H16	0.5664	0.3784	0.3761	0.022*	
C17	0.58969 (10)	0.5469 (2)	0.31504 (9)	0.0150 (4)	
C18	0.64612 (11)	0.4793 (2)	0.28280 (10)	0.0168 (4)	
H18	0.6546	0.3828	0.2909	0.020*	
C19	0.68995 (11)	0.5521 (2)	0.23897 (10)	0.0188 (4)	
H19	0.7273	0.5047	0.2163	0.023*	
C20	0.67921 (11)	0.6936 (2)	0.22840 (10)	0.0190 (4)	
H20	0.7094	0.7435	0.1987	0.023*	
C21	0.62407 (11)	0.7629 (2)	0.26133 (10)	0.0209 (4)	
H21	0.6173	0.8603	0.2547	0.025*	
C22	0.57920 (11)	0.6895 (2)	0.30369 (10)	0.0171 (4)	
H22	0.5410	0.7366	0.3252	0.020*	

### Atomic displacement parameters (Å^2^)

**Table:**

	*U*^11^	*U*^22^	*U*^33^	*U*^12^	*U*^13^	*U*^23^
I1	0.01676 (10)	0.01507 (10)	0.01419 (10)	0.00358 (4)	0.00364 (6)	0.00323 (4)
N1	0.0131 (7)	0.0105 (7)	0.0109 (7)	0.0004 (6)	0.0008 (6)	−0.0004 (6)
C1	0.0188 (11)	0.0122 (10)	0.0267 (12)	0.0042 (7)	0.0086 (9)	0.0059 (7)
C2	0.0133 (9)	0.0154 (9)	0.0113 (9)	0.0005 (7)	0.0013 (7)	−0.0029 (7)
C3	0.0151 (9)	0.0161 (9)	0.0148 (9)	0.0033 (7)	0.0025 (7)	−0.0012 (7)
C4	0.0179 (9)	0.0114 (8)	0.0145 (9)	0.0010 (7)	−0.0013 (7)	−0.0004 (7)
C5	0.0169 (9)	0.0126 (9)	0.0112 (8)	−0.0013 (7)	0.0007 (7)	−0.0001 (7)
C6	0.0133 (10)	0.0146 (9)	0.0080 (9)	−0.0014 (6)	0.0005 (7)	−0.0011 (6)
C7	0.0144 (9)	0.0119 (9)	0.0134 (9)	0.0014 (7)	0.0010 (7)	−0.0005 (7)
C8	0.0148 (10)	0.0155 (10)	0.0136 (10)	0.0006 (7)	0.0004 (8)	0.0013 (6)
C9	0.0154 (9)	0.0166 (9)	0.0098 (9)	−0.0011 (8)	0.0006 (7)	−0.0006 (7)
C10	0.0181 (10)	0.0192 (10)	0.0156 (9)	0.0010 (8)	0.0013 (7)	0.0041 (8)
C11	0.0191 (10)	0.0238 (10)	0.0145 (9)	−0.0037 (9)	0.0046 (8)	0.0016 (8)
C12	0.0150 (9)	0.0234 (10)	0.0156 (9)	−0.0009 (8)	0.0028 (7)	−0.0048 (8)
C13	0.0195 (9)	0.0175 (9)	0.0137 (9)	0.0047 (8)	0.0009 (7)	−0.0020 (7)
C14	0.0200 (10)	0.0156 (9)	0.0106 (9)	−0.0003 (8)	0.0029 (7)	−0.0003 (7)
C15	0.0223 (10)	0.0150 (9)	0.0193 (10)	0.0041 (8)	0.0063 (8)	0.0037 (8)
C16	0.0204 (10)	0.0139 (9)	0.0208 (10)	0.0013 (8)	0.0062 (8)	0.0020 (8)
C17	0.0152 (9)	0.0181 (10)	0.0117 (9)	−0.0004 (8)	0.0008 (7)	−0.0001 (7)
C18	0.0194 (10)	0.0149 (9)	0.0161 (9)	0.0012 (8)	0.0036 (7)	−0.0002 (7)
C19	0.0190 (10)	0.0222 (10)	0.0154 (9)	−0.0005 (8)	0.0056 (8)	−0.0016 (8)
C20	0.0212 (10)	0.0224 (10)	0.0133 (9)	−0.0019 (8)	0.0042 (7)	0.0035 (8)
C21	0.0257 (11)	0.0187 (10)	0.0184 (10)	0.0025 (8)	0.0022 (8)	0.0049 (8)
C22	0.0177 (9)	0.0184 (10)	0.0151 (9)	0.0042 (8)	0.0031 (7)	0.0018 (7)

### Geometric parameters (Å, °)

**Table:**

N1—C2	1.374 (2)	C11—C12	1.384 (3)
N1—C6	1.380 (2)	C11—H11	0.9500
N1—C1	1.478 (2)	C12—C13	1.394 (3)
C1—H1A	0.9800	C12—H12	0.9500
C1—H1B	0.9800	C13—C14	1.379 (3)
C1—H1C	0.9800	C13—H13	0.9500
C2—C3	1.391 (3)	C14—H14	0.9500
C2—C15	1.467 (3)	C15—C16	1.319 (3)
C3—C4	1.379 (3)	C15—H15	0.9500
C3—H3	0.9500	C16—C17	1.467 (3)
C4—C5	1.377 (3)	C16—H16	0.9500
C4—H4	0.9500	C17—C22	1.397 (3)
C5—C6	1.383 (3)	C17—C18	1.399 (3)
C5—H5	0.9500	C18—C19	1.391 (3)
C6—C7	1.461 (3)	C18—H18	0.9500
C7—C8	1.341 (3)	C19—C20	1.385 (3)
C7—H7	0.9500	C19—H19	0.9500
C8—C9	1.461 (3)	C20—C21	1.395 (3)
C8—H8	0.9500	C20—H20	0.9500
C9—C10	1.401 (3)	C21—C22	1.387 (3)
C9—C14	1.401 (3)	C21—H21	0.9500
C10—C11	1.389 (3)	C22—H22	0.9500
C10—H10	0.9500		
			
C2—N1—C6	121.83 (16)	C12—C11—C10	120.22 (19)
C2—N1—C1	120.35 (16)	C12—C11—H11	119.9
C6—N1—C1	117.78 (16)	C10—C11—H11	119.9
N1—C1—H1A	109.5	C11—C12—C13	119.12 (18)
N1—C1—H1B	109.5	C11—C12—H12	120.4
H1A—C1—H1B	109.5	C13—C12—H12	120.4
N1—C1—H1C	109.5	C14—C13—C12	121.16 (18)
H1A—C1—H1C	109.5	C14—C13—H13	119.4
H1B—C1—H1C	109.5	C12—C13—H13	119.4
N1—C2—C3	118.49 (17)	C13—C14—C9	120.16 (18)
N1—C2—C15	119.95 (17)	C13—C14—H14	119.9
C3—C2—C15	121.55 (17)	C9—C14—H14	119.9
C4—C3—C2	120.45 (18)	C16—C15—C2	123.32 (18)
C4—C3—H3	119.8	C16—C15—H15	118.3
C2—C3—H3	119.8	C2—C15—H15	118.3
C5—C4—C3	119.95 (18)	C15—C16—C17	127.73 (19)
C5—C4—H4	120.0	C15—C16—H16	116.1
C3—C4—H4	120.0	C17—C16—H16	116.1
C4—C5—C6	120.46 (17)	C22—C17—C18	118.89 (17)
C4—C5—H5	119.8	C22—C17—C16	122.98 (17)
C6—C5—H5	119.8	C18—C17—C16	118.00 (18)
N1—C6—C5	118.76 (17)	C19—C18—C17	120.53 (19)
N1—C6—C7	119.48 (16)	C19—C18—H18	119.7
C5—C6—C7	121.71 (17)	C17—C18—H18	119.7
C8—C7—C6	121.80 (18)	C20—C19—C18	120.00 (18)
C8—C7—H7	119.1	C20—C19—H19	120.0
C6—C7—H7	119.1	C18—C19—H19	120.0
C7—C8—C9	125.79 (18)	C19—C20—C21	120.00 (18)
C7—C8—H8	117.1	C19—C20—H20	120.0
C9—C8—H8	117.1	C21—C20—H20	120.0
C10—C9—C14	118.44 (18)	C22—C21—C20	119.98 (19)
C10—C9—C8	118.45 (18)	C22—C21—H21	120.0
C14—C9—C8	123.05 (17)	C20—C21—H21	120.0
C11—C10—C9	120.86 (19)	C21—C22—C17	120.56 (18)
C11—C10—H10	119.6	C21—C22—H22	119.7
C9—C10—H10	119.6	C17—C22—H22	119.7
			
C6—N1—C2—C3	−2.8 (3)	C8—C9—C10—C11	−175.20 (18)
C1—N1—C2—C3	174.87 (18)	C9—C10—C11—C12	−0.6 (3)
C6—N1—C2—C15	176.36 (17)	C10—C11—C12—C13	−1.3 (3)
C1—N1—C2—C15	−6.0 (3)	C11—C12—C13—C14	1.8 (3)
N1—C2—C3—C4	1.1 (3)	C12—C13—C14—C9	−0.2 (3)
C15—C2—C3—C4	−178.04 (18)	C10—C9—C14—C13	−1.7 (3)
C2—C3—C4—C5	1.0 (3)	C8—C9—C14—C13	175.49 (18)
C3—C4—C5—C6	−1.5 (3)	N1—C2—C15—C16	175.08 (19)
C2—N1—C6—C5	2.3 (3)	C3—C2—C15—C16	−5.8 (3)
C1—N1—C6—C5	−175.42 (18)	C2—C15—C16—C17	−177.77 (19)
C2—N1—C6—C7	−175.13 (17)	C15—C16—C17—C22	17.0 (3)
C1—N1—C6—C7	7.1 (3)	C15—C16—C17—C18	−167.2 (2)
C4—C5—C6—N1	−0.1 (3)	C22—C17—C18—C19	−1.5 (3)
C4—C5—C6—C7	177.27 (18)	C16—C17—C18—C19	−177.53 (18)
N1—C6—C7—C8	−158.92 (18)	C17—C18—C19—C20	1.9 (3)
C5—C6—C7—C8	23.7 (3)	C18—C19—C20—C21	−0.5 (3)
C6—C7—C8—C9	−173.77 (18)	C19—C20—C21—C22	−1.2 (3)
C7—C8—C9—C10	164.66 (19)	C20—C21—C22—C17	1.5 (3)
C7—C8—C9—C14	−12.5 (3)	C18—C17—C22—C21	−0.2 (3)
C14—C9—C10—C11	2.1 (3)	C16—C17—C22—C21	175.62 (19)
